# Passive Mixing Capabilities of Micro- and Nanofibres When Used in Microfluidic Systems

**DOI:** 10.3390/s16081238

**Published:** 2016-08-05

**Authors:** Lauren Matlock-Colangelo, Nicholas W. Colangelo, Christoph Fenzl, Margaret W. Frey, Antje J. Baeumner

**Affiliations:** 1 Department of Biological and Environmental Engineering, Cornell University, Ithaca, NY 14853, USA; lem44@cornell.edu; 2 Department of Radiology, Rutgers New Jersey Medical School, Newark, NJ 07103, USA; Colangnw@njms.rutgers.edu; 3 Institute for Analytical Chemistry, Chemo- and Biosensors, University of Regensburg, Regensburg 93040, Germany; Christoph.Fenzl@chemie.uni-regensburg.de; 4 Department of Fibre Science and Apparel Design, Cornell University, Ithaca, NY 14853, USA; margaret.frey@cornell.edu

**Keywords:** nanofibres, fluid mixing, microfluidics, biosensors

## Abstract

Nanofibres are increasingly being used in the field of bioanalytics due to their large surface-area-to-volume ratios and easy-to-functionalize surfaces. To date, nanofibres have been studied as effective filters, concentrators, and immobilization matrices within microfluidic devices. In addition, they are frequently used as optical and electrochemical transduction materials. In this work, we demonstrate that electrospun nanofibre mats cause appreciable passive mixing and therefore provide dual functionality when incorporated within microfluidic systems. Specifically, electrospun nanofibre mats were integrated into Y-shaped poly(methyl methacrylate) microchannels and the degree of mixing was quantified using fluorescence microscopy and ImageJ analysis. The degree of mixing afforded in relationship to fibre diameter, mat height, and mat length was studied. We observed that the most mixing was caused by small diameter PVA nanofibres (450–550 nm in diameter), producing up to 71% mixing at the microchannel outlet, compared to up to 51% with polystyrene microfibres (0.8–2.7 μm in diameter) and 29% mixing in control channels containing no fibres. The mixing afforded by the PVA nanofibres is caused by significant inhomogeneity in pore size and distribution leading to percolation. As expected, within all the studies, fluid mixing increased with fibre mat height, which corresponds to the vertical space of the microchannel occupied by the fibre mats. Doubling the height of the fibre mat led to an average increase in mixing of 14% for the PVA nanofibres and 8% for the PS microfibres. Overall, mixing was independent of the length of the fibre mat used (3–10 mm), suggesting that most mixing occurs as fluid enters and exits the fibre mat. The mixing effects observed within the fibre mats were comparable to or better than many passive mixers reported in literature. Since the nanofibre mats can be further functionalized to couple analyte concentration, immobilization, and detection with enhanced fluid mixing, they are a promising nanomaterial providing dual-functionality within lab-on-a-chip devices.

## 1. Introduction

Electrospinning is a well-understood and simple fibre fabrication process in which an electric field is applied to a polymer spinning dope to generate nano- and microscale fibres [1]. The nonwoven fibre mats produced by electrospinning are characterized by extremely large surface-area-to-volume ratios, high porosities, and small pore sizes. Additionally, fibres can be spun from a wide range of materials, including biocompatible polymers, and are easily functionalized through the incorporation of useful materials into the spinning dope or by post-spinning immobilization techniques [2].

Electrospun fibres have been used in a variety of applications, including the development of tissue engineering scaffolds [3,4,5,6], sensors [7,8,9,10,11], high-performance batteries [12,13,14], and vehicles for drug delivery [15,16]. Several review articles have been written about the many promising uses of electrospun nanofibers [17,18,19,20]. In particular, the incredibly high surface area afforded by electrospun fibre mats has been utilized to improve the performance of a wide range of analytical devices [2,21,22]. Fibre-based sensors feature an increased number of binding sites and faster mass transfer rates than conventional sensors, resulting in much lower limits of detection and faster analyte detection [22]. Recently, electrospun nanofibres have been incorporated into microfluidic sensing systems to allow for enhanced sample preparation and analyte detection [7,23,24,25,26,27]. In some applications, water-soluble nanofibers have been used to facilitate on-chip reagent storage in microfluidic biosensors [7,27]. Jin et al. used electrospinning to encapsulate a horseradish peroxidase tagged antibody into polyvinylpyrrolidone nanofibers [7]. Encapsulating the antibodies within the water-soluble fibres allowed for stable, long-term storage of the biorecognition element on-chip. This approach has also been used to allow on-chip enzyme storage [27]. Further, non-water-soluble electrospun nanofibers have been used as a substrate for microfluidic HIV immunoassays [26], as on-chip sample concentrators [23], and as a substrate for microfluidic *E.coli* detection [25]. In these applications, the non-water-soluble nanofibres were able to withstand the fluid flow within the channels and dramatically increased the functional surface area available within the devices. However, to date, the effects of electrospun fibre mats on the fluid flow within these microfluidic systems has not been studied.

Microfluidic channels generally feature low Reynold’s number fluid flow, resulting in laminar flow patterns and limited fluid mixing [28,29,30]. Achieving rapid and reliable fluid mixing is essential for facilitating (bio)chemical reactions and allowing for adequate access of analytes to functional surfaces within microfluidic sensors [28]. While some applications allow for pre-mixing of reagents prior to injection into the microfluidic device, often it is necessary for mixing to occur on-chip [29]. Rapid chemical reactions that take place on a sub-millisecond time scale, such as protein folding, are often studied using microfluidic devices and require on-chip reagent mixing [31]. Additionally, microfluidic devices are well-suited for performing chemical reactions with hazardous or expensive chemicals because of the small internal volume of the devices and relative safety compared to bench-top reactions [29]. Finally, on-chip mixing is crucial to the success of microfluidic assays, both to facilitate access of analytes to all the functional sites within the sensor and to allow for multiple-step assays [32,33]. In general, mixing within microfluidic channels can be improved through increasing the contact surface and lowering the diffusion path between fluids [28,34].

Many micromixers have been proposed and developed to address this need for fluid mixing in miniaturized devices [35,36,37]. These mixers can either be integrated directly within the microfluidic channel or function as a separate component that interfaces with the microfluidic device. Mixers are classified either as passive or active mixers [28,29]. Passive mixers utilize diffusion and chaotic advection to achieve mixing [29,30]. Active mixers, on the other hand, apply an external energy field to enhance mixing within the channel [29]. In general, active mixers produce reliable, complete mixing but are more complicated and expensive than passive mixers due to the need to integrate the external force field [28]. Therefore, passive mixers are often much simpler than active mixers and can be easier to fabricate and utilize. Several common passive mixers include T-type, Y-type, split and recombine, multi-laminating and jet colliding mixers [29]. In each of these mixers, the flow pattern is manipulated by different channel geometries or by incorporating microstructures within the channel. Furthermore, in order for analyte detection to be performed within the microchannel, additional modifications must be made to immobilize the biorecognition element on the channel surfaces. Currently, most mixers are fabricated using photolithography or micromachining, which require access to a cleanroom and/or expensive machinery [38].

Electrospun fibres, on the other hand, are relatively easy to fabricate and do not require a cleanroom. A typical electrospinning apparatus consists of a high voltage power source, a syringe pump, a spinneret (typically a syringe) filled with a polymer spinning dope, and a grounded collector plate (Figure S1) [1,2]. During electrospinning, the viscous polymer solution is slowly pumped out of the spinneret, which is placed across from the grounded collector plate. When voltage is applied to the spinning solution, it forms a Taylor cone at the tip of the spinneret [2]. Once the electrostatic attraction between the charged polymer solution and the grounded collector plate overcomes the surface tension at the tip of the spinneret, fibres will accelerate from the tip of the Taylor cone and collect on the grounded collector plate [1]. Previously, we have demonstrated that electrospun poly(vinyl alcohol) (PVA) nanofibres can be used for sample purification and analyte concentration within microfluidic systems [23]. However, due to their complex nonwoven three-dimensional structure, it is hypothesized that electrospun fibre mats can also be used to enhance fluid mixing within microfluidic channels through manipulation of the flow patterns within the mats. Additionally, in contrast to micromixers that utilize channel geometry or two-dimensional microstructures, fibre-based mixers can be fabricated directly from spinning dopes functionalized with a wide range of additives such as conductive materials [39,40], enzymes [41], biotin [42,43], and aptamers [44]. These functionalized fibres could be used to perform analyte detection and fluid mixing without requiring further modifications of the microchannel. In this work, two different polymers, PVA and PS, were used to produce fibres with different diameters and morphologies to determine their effect on mixing within PMMA microfluidic channels. The work here was done using a Y-shape design, however, nanofiber mats can be introduced into any microchannel system as long as the height and width allow for successful bonding of the device while the nanofiber mats are within the channels.

## 2. Materials and Methods

### 2.1. Electrospinning

The PS fibres were spun using a Spraybase vertical electrospinning system (Avectas, Dublin, Ireland), while the PVA nanofibres were spun using a homemade horizontal electrospinning system as previously described [23,24]. Positively charged PVA fibres were produced by adding hexadimethrine bromide (polybrene, Sigma-Aldrich, St. Louis, MO, USA) into the PVA spinning dope. The PVA used had a molecular weight of 78,000 g/mol and is 99.7% hydrolyzed (Polysciences Inc., Warrington, PA, USA) [24]. A 10% w/v PVA spinning dope was first created by dissolving PVA in deionized (DI) water at 95 °C for four hours. Then, polybrene was dissolved in DI water at room temperature for 10 min, and was added to the PVA solution to make a final spinning dope with a 90/10 w/w PVA/polybrene ratio. The nonionic surfactant Triton X-100 was added to the spinning dope to improve spinnability, and the resulting solution was mixed for two minutes using a vortex at its highest speed. The spinning dope was then loaded into a 5 mL BD plastic syringe with an 18 gauge needle, which was placed horizontally into a syringe pump. The syringe pump was set to 0.01 mL/min. A high voltage power source was connected to the spinning needle and set to 15 kV (Gamma High Voltage Research Inc., Ormond Beach, FL, USA). A piece of copper wrapped in aluminum foil was used as a grounded collector plate and was placed 15 cm from the syringe tip. After spinning, the aluminum foil was removed from the copper plate and was cut into strips using a razor blade. The nanofibre mats were then peeled off the foil strips using tweezers and were placed on pieces of poly(methyl methacrylate) (PMMA) that had undergone treatment in an UV-ozone (UVO) oven for 15 min (Jelight Company Inc., Irvine, CA, USA). The UVO oven contained a low pressure mercury vapor grid lamp, which had an output of 28,000 mW/cm^2^ at 254 nm. During UVO treatment, oxygen was flowed through the oven at 0.5 L/min. The PVA fibres were initially spun as 20, 30, and 40 µm thick mats, however the nanofibre distribution varied along the heights of the mats, producing inconsistent mixing within the channels. Therefore, nanofibre mats of approximately 10 µm height were spun and stacked to create thicker nanofibre mats with more uniform nanofibre distributions. The mats were cut into 3 mm, 5 mm, and 10 mm long strips for the studies examining the effect of nanofibre mat length on mixing.

The PS microfibres were spun using a solvent solution composed of 50/50 v/v tetrahydrofuran/dimethylformamide. Polystyrene with a molecular weight of 280,000 g/mol (Sigma-Aldrich) was dissolved in the solvent solution at room temperature for 24 h on a stir plate on its medium setting. Different fibre morphologies were produced by using three different PS concentrations in the spinning solutions: 12.5%, 15%, and 17.5% w/v.

The PS spinning solution was loaded into a 5 mL glass syringe (BD) and was spun using a Spraybase vertical electrospinning system with an 18 gauge needle. The fibres were spun using a 20 cm vertical distance, 10.6 kV applied voltage, a 0.01 mL/min flow rate, and a circular metal collector plate. The fibres were then manually transferred to PMMA squares that had been treated in the UVO oven for 4 min at an oxygen flow rate of 0.5 L/min and lamp power of 28,000 mW/cm^2^ (Jelight Company Inc.). The fibre mats were cut into 10 mm long strips using a scalpel and unwanted fibres on the PMMA surfaces were removed using a strip of adhesive tape. The mats were incorporated onto the PMMA chips in one-layer or two-layer configurations.

### 2.2. Analysis of Fibre Morphology

The PVA nanofibre mats were assessed using a SP2 confocal microscope (Leica, Wetzlar, Germany) to measure the thickness of the mats prior to incorporation into the microfluidic channels [23]. The polybrene/PVA nanofibres produced in our lab have previously been demonstrated to have an average diameter between 450 and 550 nm [24].

**Figure 1:**
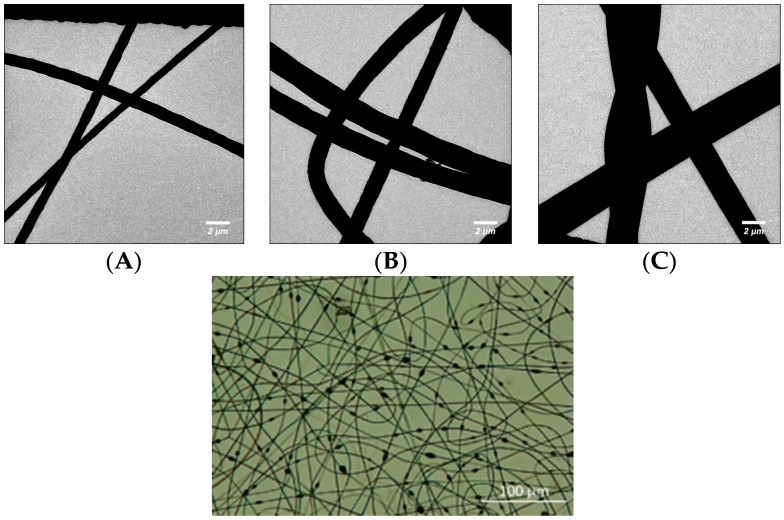
(**Top**) TEM image of (**A**) 12.5% w/v (**B**) 15% w/v and (**C**) 17.5% w/v PS microfibres. Fibres were spun onto carbon-coated grids for 15 s. Micrographs taken using a type CM 12 Philips TEM at 120 kV; (**Bottom**) Morphology of 12.5% w/v PS microfibres. Fibres were spun onto a metal collector plate and transferred to a UVO-treated piece of PMMA. Image taken with a Nikon Digital Eclipse C1 confocal microscope in brightfield setting.

Micrographs of the three different types of PS microfibres were taken using a transmission electron microscope (TEM, type CM 12 from Philips, Hamburg, Germany) at 120 kV and a magnification ranging from 800-fold. In order to facilitate TEM analysis, the fibres were spun directly onto carbon-coated copper grids (400 mesh from Plano, Wetzlar, Germany) for 15 s. The diameter of the cylindrical portion of the different nanofibre types was measured in six points per fibre type and averaged. The 12.5% w/v PS fibres had a 0.8 ± 0.14 µm diameter, 15% w/v PS fibres had a 1.5 ± 0.2 µm diameter, and 17.5% w/v PS fibres had a 2.7 ± 0.5 µm diameter (Figure 1). Additionally, the 12.5% w/v PS fibres had a heavily beaded morphology (Figure 1), the 15% w/v fibres had sporadic beading, and the 17.5% w/v had large cylindrical diameters with no beads. The beads were not considered when measuring the microfibre diameters.

### 2.3. Microchannel Fabrication

Y-shaped microfluidic channels were stamped into PMMA using hot embossing with a copper template (Figure S2) [24]. The Y-shaped channel was used to allow injection of two different fluid solutions into the microchannel in order to simplify visualization of fluid mixing within the channel. The copper template was fabricated at the Cornell Nanoscale Facility (CNF) using KMPR 1050 photoresist (MicroChem Corp., Westborough, MA, USA) and copper electroplating to produce raised copper channels on a smooth copper plate [45]. The channels used in this study were 31 µm deep, 1 mm wide, and 29 mm long. The channels used with the PVA nanofibres were embossed using a Carver Laboratory Hot Press (Carver, Inc., Wabash, IN, USA) at 130 °C and 10,000 lbs (44,482 N) of force. The channels used with the PS microfibres were embossed using a Specac Hot Press (Specac Ltd, Orpington, Kent, UK) at 100 °C and 0.1 ton of force for 5 min. The inlet and outlet holes were drilled at each end of the channel with a 0.8 mm steel drill bit.

UVO-assisted thermal bonding was used to create the completed microfluidic devices [23]. First, the pieces of PMMA that had been embossed with the microfluidic channels were UVO treated using an oxygen flow rate of 0.5 L/min and lamp power of 28,000 mW/cm^2^. The PMMA channels used with the PS microfibres was treated for 4 min, while the PMMA channels used with the PVA nanofibres was treated for 15 min. Then, the pieces of PMMA with the fibre mats were UVO treated for 4 min. The two pieces of PMMA were sandwiched together so that the fibres faced the microchannels. The sandwich assembly was placed between two blank pieces of copper and pressed on the hot press. PS fibres were bonded into channels at 80 °C and 0.1 ton force, while PVA nanofibres were bonded into channels at 100 °C and 10,000 lbs (44,482 N) of force. The different UVO treatment times, temperatures and applied forces were necessary due to differences between the two hot presses used. Polyvinyl chloride tubing (Tygon, Saint-Gobain Performance Plastics, Paris, France) with a 0.508 mm external diameter was glued into the inlet and outlet holes with Quicktite instant adhesive gel (Loctite, Henkel Corporation, Westlake, OH, USA).

### 2.4. Fluid Mixing

**Figure 2:**
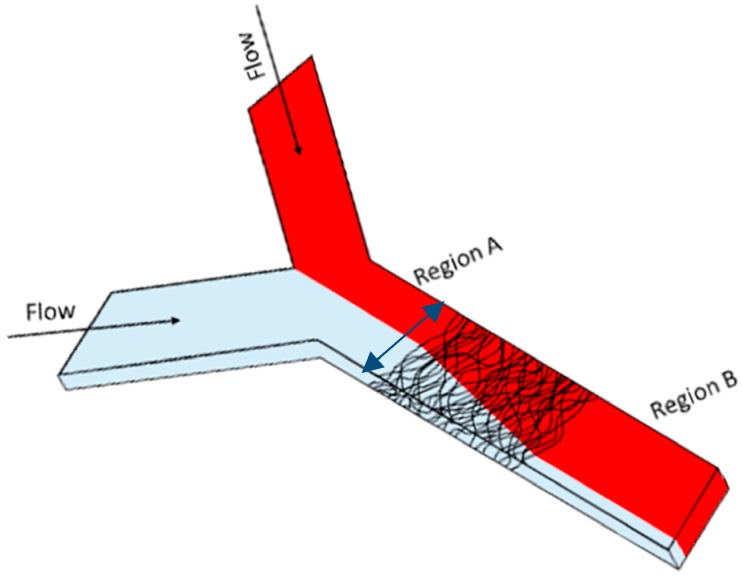
Angled top view of the Y-channel mixer with embedded nanofiber mats. One inlet was used to fill the channel with DI water, the other filled the channel with SRB in water. Nanofibre mats with different lengths and thicknesses were placed in the center of the channel to encourage fluid mixing. The extent of fluid mixing in Region A of the channel (before fibre mat) was compared to the extent of fluid mixing in Region B (after fibre mat) using ImageJ to measure how the pixel intensity changed along a 1 mm wide vertical column that spanned the channel (blue arrow in picture).

Each Y-shaped microfluidic channel was filled with water in one inlet and a 0.03 M sulforhodamine B (SRB) in water solution in the other inlet (Figure 2). 

Because channels filled with nanofibre mats are prone to form air bubbles at low flow rates, each channel was initially filled with fluid at 20 µL/min for 5 min to remove air bubbles from the nanofibre mats and ensure that they didn’t impact the mixing observed. Then, the flow rate was dropped to 5 µL/min and a steady flow profile was allowed to develop for 5 min before a fluorescent microscopy image was taken of the channel (Leica). This was repeated for flow rates between 4 µL/min and 1 µL/min. Lower flow rates were not used as pulsatile flow was observed at rates below 1 µL/min.

Several fluid mixing experiments were recorded using an Eclipse 90i confocal microscope (Nikon, Tokyo, Japan) to confirm that the observed mixing was consistent along the height (z direction) of the channel (Figure S3 in Supplementary Information). The fluid mixing experiments were conducted on the stage of the confocal microscope. During fluid flow, the confocal was used to perform a z-scan of the channel in order to examine the fluid flow profile along the z direction of the channel. Images of the channels were taken at 1 µm intervals in the z direction.

### 2.5. Data Analysis

ImageJ was used to measure the pixel intensity of each pixel in a column along the 1 mm width of the channel (Supplementary Information). In control channels, the pixel intensities along this column will vary from high values (in SRB portion of channel) to low values (in water portion of the channel) (Figure 2). Well-mixed channels will have uniform pixel intensities along the column, as the entire channel is filled with the same fluid mixture. The mixing index of each column of pixels was determined using the following formula [46,47]:

$$mixing\;index=\sqrt{\frac{1}{N}\sum^{\text{​}}{\left(\frac{I_{K}-I_{O}}{I_{O}}\right)}^{2}}$$

where *I_K_* = intensity of a single pixel in a column; *I_0_*= average intensity of all pixels in a column; *N* = number of pixels in a column.

The average mixing index at the inlet of each channel was determined by averaging the mixing index of each column in a 50 pixel wide section of the channel before the nanofibre mats where a steady flow profile existed (region A in Figure 2). The average mixing index of the channel after the nanofibre mat was determined by averaging the mixing index of each column in a 50 pixel wide section of the channel near the outlet of the channel where again a steady flow profile existed (region B in Figure 2). Each set of parameters was tested using between 3 and 5 different channels to determine reproducibility of the results.

### 2.6. Statistics

Average Mixing Index data for all the channels were compiled in Microsoft Excel (Microsoft, Redmond, WA, USA). A MATLAB (MathWorks, Natick, MA, USA) code was written to perform multiple linear regression analysis on the data and determine the statistical significance of mixing within channels containing the nanofibre mats (as compared to mats containing no nanofibres) (Supplementary Information Code S1, Code S2, Code S3, and Code S4). Multiple comparisons were made using the Holm test, which controls for the accumulation of error that occurs when multiple t tests are performed [48]. The Holm test sequentially compares the p values of multiple comparisons (from smallest p value to largest p value), with each comparison getting progressively less conservative to account for the number of comparisons that have already been done [49]. The roles of nanofibre mat length, nanofibre mat height (i.e., number of fibre layers), and flow rate on observed fluid mixing was analyzed for the PVA nanofibres. The influence of nanofibre mat morphology (diameter and number of fibre layers) and flow rate on observed fluid mixing was determined for the PS microfibres. Each condition was run in at least triplicate and the mixing values used by the MATLAB code were the average of 50 pixel-wide columns. Significance was set as p < 0.05.

## 3. Results

### 3.1. Thick Nanofibre Mats

Initially, PVA nanofibre mats that were 20, 30 or 40 µm thick were incorporated into the microfluidic channels (31 µm deep) to determine if thicker nanofibre mats produced better mixing by increasing the volume of the channel occupied by nanofibres. Each nanofibre thickness was tested as a 3 mm, a 5 mm, and a 10 mm wide strip of fibres. While mixing was indeed observed in many of the channels tested, there was significant variability in the flow profiles produced from the same nanofibre mat morphology (Figure S4 in Supplementary Information). Confocal microscopy of the nanofibre mats demonstrated that the nanofibre distribution along the thickness of the mats was not uniform. The mats were most dense at the bottom of the mat and frequently had a sparse nanofibre distribution in the top of the mat (Figure S4 in Supplementary Information). However, while the fibre distribution between nanofibre mats with the same thickness also varied significantly along their depths, the first ~10 µm showed a consistent morphology both within a single nanofibre mat and between different fibre mats.

Several groups have previously demonstrated that electrospun nanofibres are not evenly collected on the grounded collector plate due to the shape of the electric field that exists between the charged spinneret and the collector plate [50,51,52]. During electrospinning, the thickness uniformity of the nanofibre mats has been shown to decrease with longer spinning times, with short spinning times yielding the most uniform nanofibre mats [50]. Therefore, it was determined that the variations seen in the flow profiles in the thick nanofibre mats were caused by these non-uniformities.

### 3.2. Layered PVA Nanofibre Mats

**Figure 3:**
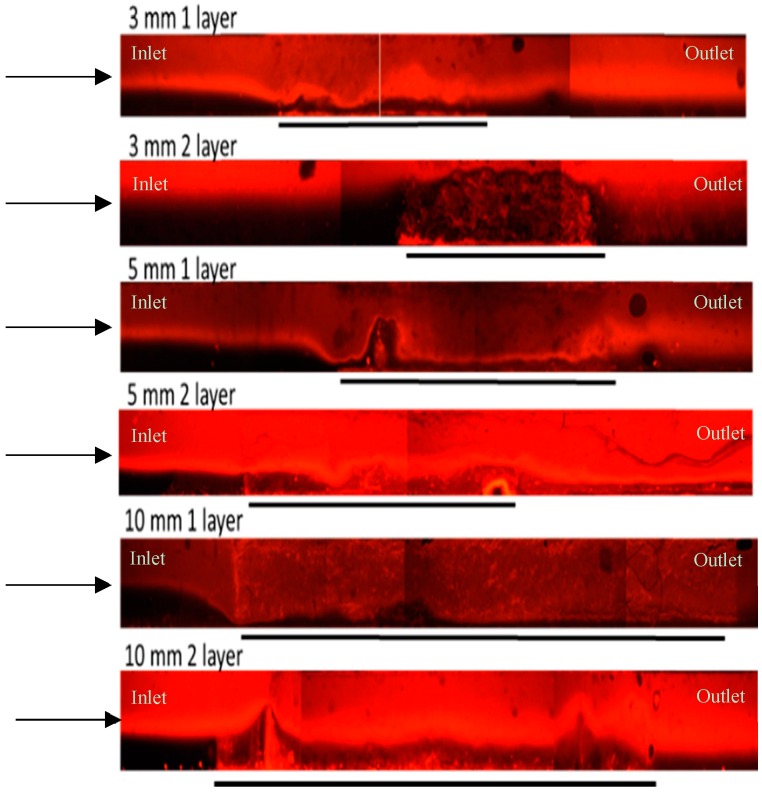
Example images of fluid flow through nanofibre mats within microfluidic channels. Each set of nanofibre mat morphologies was investigated at a flow rate of 1 µL/min. Arrows indicate direction of fluid flow through the channels. Black lines indicate the location and length of the nanofibre mats within the channels. The inlet of each channel consisted of a red fluid stream (SRB) and a black fluid stream (water). After the nanofiber mats, the two fiber steams have mixed, producing a more homogeneous diluted SRB/water outlet fluid solution. Fluorescent microscope, 5× objective.

In order to address the need for nanofibre filters with uniform morphologies, Zhang et al. [50] and Podgórski et al. [52] created multi-layered nanofibre mats by stacking thin nanofibre mats. These multi-layered filters demonstrated both more uniform fibre distribution and improved filtration performance. Therefore, to improve the reproducibility of our mats, we spun ~10 µm thick nanofibre layers and stacked them to obtain thicker nanofibre mats with more uniform fibre distributions. This layered approach was used with varying fibre mat length (3, 5 and 10 mm) and thickness (one layer or two layer). Images taken of the different channels during fluid flow demonstrated that the mixing produced by all of the nanofibre mat morphologies was nearly identical (Figure 3).

**Figure 4:**
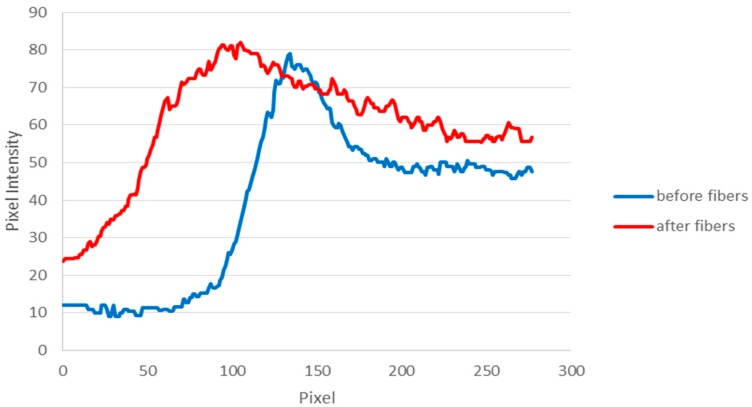
Pixel intensities of a region before the nanofibre mat and after the nanofibre mat for a 5mm, two-layer mat with flow rate of 1 µL/min. Pixel intensity values shown represent the average value of 50 vertical columns across the 1 mm width of the channel.

For data analysis, ImageJ (U.S. National Institutes of Health, Bethesda, MD, USA) was used to measure the pixel intensity of each pixel in a vertical column that spanned the 1 mm width of the channel (Figure 2). In the unmixed regions before the nanofibre mats, the pixel intensities along a column had a binary profile, indicating that the channel was divided into two different flows that were not mixed (Figure 4). This is expected for laminar flow patterns. The half of the channel filled with water had a measured pixel intensity of ~10, the half of the channel filled with SRB had a measured pixel intensity of ~50. A peak was observed at the interface of the two solutions. As the SRB concentration chosen exhibited a slight quenching effect, its diluted form at the interface therefore results in a higher fluorescence signal. This two-phase system enters the nanofibre mats. Mixing occurring within the nanofibre mat will result in a decline of the signal difference observed between the two halves of the channel. For quantification, the pixel intensity distribution is also measured after the solution exits the nanofibre mat. As can be seen in Figure 4, the profile becomes more uniform, with the pixel intensity increasing rapidly and plateauing where the solution in the channel is mixed. Furthermore, it can be observed that a pure buffer solution is no longer present as the minimum pixel intensity within the channel increased significantly to ~25. The highest pixel intensity is reached more quickly in the region after the nanofibres and is more consistent, as the SRB dye has spread to a larger portion of the channel and has mixed into a more uniform profile.

The average mixing index at the inlet and outlet of each type of channel was calculated using ImageJ (Table 1). The change in outlet mixing index between the control channels (no nanofibres) and the nanofibre channels was calculated to determine whether the channels containing nanofibre mats exhibited more fluid mixing than empty control channels (Table 1 and Table S1 in Supplementary Information). Based on their outlet mixing indexes, the nanofibre mats studied produced the most mixing in the two-layer mat configuration (Table 1). As the one-layer mat will only occupy approximately one third of the total height of the microchannel, the fluid can partly pass above the nanofibre mat without having to flow directly through the mat. On the other hand, the two-layer mat will fill most of the channel height, forcing the fluid to pass through the nanofibre mats. Additionally, the outlet mixing index values for the two-layer nanofibre mats had lower standard deviations than the one-layer mats or the control channels, indicating that the mixing observed in the two-layer mat morphology is more reproducible.

It was also observed that for the two-layer mat morphologies, the average mixing index at the inlet of the channels was markedly lower than the inlet mixing index of the channels containing no nanofibres (Table 1). We assume that this is caused by back pressure produced by the mats. In turn, this pressure would slow the fluid velocity at the inlet of the channel, allowing for more diffusive mixing before the fluid even enters the nanofibre mat and enhancing the overall desired mixing of the two solutions.

The effect of fluid flow rate on observed mixing was also determined. As expected, the amount of mixing observed in the channels increased slightly as the flow rate decreased (Figure S5 and Table S2 in Supplementary Information).

Multiple linear regression was used to determine if the mixing observed in the channels containing PVA nanofibres was statistically different from the diffusive mixing observed in empty control channels (Table S1 in Supplementary Information). The analysis showed that the increase in fluid mixing observed in all the PVA nanofibre mats was statistically significant (p < 0.05), with the greatest increase in mixing observed with the two-layer mat morphologies as expected. The p value for mixing observed in the 3 mm 2 layer morphology is 2.35 × 10^−10^ with values below 0.05 generally considered to show statistically significant difference in data sets [53].

In order to better understand the influence of nanofibre mat height (number of layers) and length on the observed mixing, additional statistical analysis was performed. Based on the outlet mixing index values for the channels containing PVA nanofibre mats, the number of fibre mats and the flow rate appear to have an effect on the mixing produced in the microchannels, while the length of the nanofibre mat does not appear to play a role. Therefore, multiple linear regression was used to test these hypotheses with significance set as p < 0.05 (Table S2 in Supplementary Information). Overall, the hypothesis that the number of fibre mats affects the mixing index was highly significant (p = 5.15 × 10^−8^). On average, the two-layer mats reduced the mixing index by 0.14 relative to the one-layer mat. The flow rate was also found to play a statistically significant role in the observed mixing, with p = 0.0045. The mixing index increased by an average of 0.03 for each 1 µL/min increase in flow rate. However, the effect of nanofibre mat length was not significant (p = 0.24).

### 3.3. Layered PS Microfibre Mats

In order to study the effects of fibre diameter and shape on fluid mixing within the channels, PS microfibres of different diameters were incorporated into Y-shaped channels (Figure S6 in Supplementary Information). The PS fibres were chosen for this study since they afforded a much larger diameter (2–7 times lager) than the positively charged PVA nanofibres used in the previous parts of this work. Several labs have successfully used micropillars with diameters in the 10 µm range to encourage fluid mixing within microfluidic systems [54,55]. Therefore, investigation of mixing within the PS mats would determine if fibres with micrometer diameters could similarly be used as obstacles to enhance fluid mixing. Fibres with diameters larger than 2.7 µm were not investigated as they could not be successfully bonded into the microfluidic channels. Additionally, the PS fibres have a beads-on-a-string morphology when spun at lower polymer concentrations and a smooth, cylindrical morphology at high concentrations. Therefore, PS fibres were also used to determine which fibre shape (beaded or smooth) produces more mixing within the channels. In the end, three different PS fibre types were used in this study (Figure 1). Fibres spun from a 12.5% w/v PS solution had a beads-on-a-string morphology and a diameter of 0.8 ± 0.14 µm. Fibres spun from a 15% w/v PS solution have few beads and a smooth cylindrical morphology with a diameter of 1.5 ± 0.2 µm. Finally, fibres spun from a 17.5% w/v PS solution have no beads and a cylindrical morphology with a diameter of 2.7 ± 0.5 μm.

Based on the previous results, which indicated that mat length does not play a significant role in mixing, the microfibre mats were always 10 mm long and stacked in one or two layers within the channels. The 10 mm mat length was chosen as PS fibres are difficult to cut into smaller mats and the most reproducible mats could be made at this length as shorter mats were prone to tearing while being cut. SRB and water were pumped through the channels at flow rates between 1 µL/min and 5 µL/min (Figure S6 in Supplementary Information). Similar flow profiles at the inlet (Figure S7 in Supplementary Information) and outlet (Figure S8 in Supplementary Information) of the channels were observed for the PS systems as described above for the PVA nanofibre mats.

The average mixing index values at the inlets and outlets of the different channels were calculated using ImageJ (Table 2). The difference in outlet mixing index for the microfibre channels and the control channels was used as a metric of how the various PS fibre morphologies affected mixing within the channels.

The mixing in the PS fibres exhibited many of the same trends as the mixing in the PVA nanofibre mats, though the average mixing indexes were higher for the PS fibres (indicating less mixing). As with the PVA nanofibres, the two-layer PS fibre morphologies produced a larger change in outlet mixing index than the one-layer morphologies (Table 2). Additionally, the two-layer morphologies had smaller standard deviations in their outlet mixing index values, indicating that their mixing is more reproducible than the one-layer mats. Finally, the inlet mixing index values for the two-layer mats were also smaller than the inlet mixing index of the control channels, indicating that the increase in mixing observed in two-layer fibre mats is due both to an increase of diffusive mixing in the inlet region of the channel and mixing from fluid flow through the fibre mat itself. The PS fibres produced a smaller change in outlet mixing index than the PVA nanofibres, indicating that it is was more effective to use the nanoscale fibres than the larger microfibres to induce mixing in the microfluidic channels.

In order to determine if the mixing in the different PS mats was statistically significant (when compared to empty control channels), multiple linear regression was used. Multiple comparisons within the mat type group were made using the Holm test with the overall p-value set as p < 0.05. The different fibre weight percentages and layer combinations were represented as individual variables and compared for significance relative to their respective control of no fibre mat (Table S3 in Supplementary Information).

There were three PS microfibre morphologies that produced a statistically significant increase in mixing when compared to empty control channels: 1 layer 17.5% w/v PS, 2 layer 15% w/v PS, and 2 layer 17.5% w/v PS. Therefore, though overall mixing was highest with PVA nanofibre mats (which have the smallest diameters of all the fibres used in this study), within the PS microfibres mixing increased with increased diameter. This suggests that the mixing observed in the microfibres may be caused by a different mechanism than the mixing observed in the nanofibre mats. Multiple linear regression analysis was performed once more to determine the effect of fibre mat thickness (number of layers) and morphology (12.5%, 15%, or 17.5% w/v PS) on the mixing observed in the PS mats (Table S4 in Supplementary Information). Once again, the fibre mat thickness had a very significant effect on fluid mixing, though the magnitude of this effect was decreased for the PS fibres (−0.084 change to the outlet mixing index) when compared to using two layers of PVA fibres (−0.142 change to the outlet mixing index). Additionally, increasing the diameter of PS fibres also increased the mixing observed in the channels. Finally, flow rate once again affected the mixing observed, with decreasing mixing with increasing flow rate (Table S4).

## 4. Discussion

Microfluidic analytical systems depend on efficient fluid mixing to ensure that the desired reactions can take place under optimal conditions. This work examines the mixing effect caused by nanofibre mats that have been integrated into a Y-shaped polymer microchannel (Figure 2). The question at hand is whether electrospun fibre mats used primarily for immobilization, separation, or transduction purposes also afford enough mixing capabilities so that the additional integration of passive or active mixers into a microanalytical system is unnecessary. Many passive micromixers reported in the literature utilize obstacles to split inlet streams into narrow flow streams, thus increasing the interfacial areas of the fluids and decreasing mixing time [56,57]. The obstacles used within these studies all have diameters on the order of 10 or 100 µm, making them substantially larger than the fibres used in this study [28,29,35,57]. Indeed, the individual fibres themselves are likely too small to serve as obstacles that redirect the flow pattern within the channels. However, it was postulated that the dense, porous structure of the fibre mats would lead to enhanced mixing of solutions flowing through the mats. In order to investigate which type of fibrous media was most capable of causing increased mixing within microfluidic channels, both electrospun nanofibres (PVA) and small electrospun microfibres (PS) were incorporated into the microchannels. Parameters investigated were fibre mat density, mat thickness (these two are closely related in the electrospinning process) [23], mat length, fibre diameter, fibre shape, and flow rate.

### 4.1. Comparison of PVA and PS Fibre Mixing 

The amount of fluid mixing observed was highest in the PVA nanofibre mats, which had a much smaller diameter (450–550 nm) than the PS microfibres (0.8 µm, 1.5 µm, and 2.7 µm). However, within the PS microfibre mats, mixing increased with increasing fibre diameter, indicating that the mechanism of mixing within the PS and PVA fibres is different.

We assume that the largest PS microfibres can cause some flow manipulation through the volumetric presence of the microfibre, similar to using micropillar obstacles within the channel. However, the microfibres used within this study are still much smaller than the 10–100 µm diameter obstacles frequently used within micromixers, resulting in less mixing than reported in other obstacle-based micromixers (Table 3). In contrast, with the PVA nanofibre mat, it is the porous, inhomogeneous 3D shape of the mat itself that causes the increased dispersive mixing. It has been reported that micromixers that utilize an asymmetric arrangement of obstacles yield significantly more mixing than a symmetric obstacle arrangement [34,57]. An asymmetrical obstacle distribution within the channel results in different resistances to flow in the lateral direction of the channel, causing the fluids to find paths of least resistance through the obstacles [34]. The fluid flow is then repeatedly distorted and redirected as it flows through the obstacles, which in turn increases mixing [34]. The inhomogeneous pore size, pore density and fibre distribution of the PVA nanofibre mats similarly force the fluid to find paths of least resistance as it enters and travels through the fibre mat, producing the increase in mixing reported in this work. On the other hand, the PS microfibre mats used in this study have a more homogeneous pore size and distribution within the channels and thus produce less mixing within the channels than the PVA nanofibre mats. While the average pore size of the mats used in this study was not measured in order to prevent damage to the mats prior to incorporation within the microfluidic devices, average pore size within nanofibre mats typically correlates with the nanofiber diameter [58]. Our lab has previously measured the mean pore size of non-layered PVA mats using an 1100-AEHXL capillary flow porometer. The PVA mats had a measured mean pore size between 0.62 and 0.72 μm, which agrees with predictions for nanofibre mats with diameters of 300–550 nm [58]. Because fibre mat pore size and distribution appear to have a significant effect on mixing, future studies will look at optimizing pore size and distribution within the fibre-based micromixers. Several labs have reported methods for controlling the size, shape, and distribution of electrospun nanofibre pores, which could be used to examine the relationship between mat porosity and fluid mixing [59,60,61,62].

The chemistry of the two polymers used likely also contributed to their different mixing behaviours. The hydrophilicity of PVA [63] may facilitate movement of water into the pores of the PVA fibre mats, thus producing the increased mixing observed. Further, the hydrophobic PS fibres [64] would likely have repelled the mixing fluids, making it more difficult for fluid to fill the pores of the PS fibre mats and mix together.

For all the fibres studied, mixing increased with mat height and with decreasing flow rate (Table S2 in Supplementary Information). Additionally, the fibre mat length did not have a significant impact on the final mixing observed, which leads to the conclusion that, for mixing in the PVA fibre mats, most of the observed mixing effects are from the fluid entering and exiting the fibre mats. Finally, the mixing index within the channels containing two layers of PS or PVA fibre mat was lower than the control mixing index at both the inlet and outlet of the channels, suggesting that the observed mixing was also caused by increased back pressure in the channels containing fibre mats.

### 4.2. Comparison to Conventional Micromixers

To date, passive fluid mixing in microfluidic channels is typically accomplished through patterning of microstructures into polymer channels or by creating complex channel geometries that alter the flow pattern [29]. While these passive mixers can be very effective, they often utilize multi-step lithography or require aligned assembly of multilayered channel geometries [57]. Additionally, simple channel geometries such as T-junction and Y-junction channels have also been used to allow for complete diffusive mixing by optimizing channel length [67,68]. While this can be very effective for thin microchannel designs, the length of channel required to allow for mixing in these simple channel designs is dependent on channel width and mixer aspect ratio [69,70], with wider channel designs requiring channel lengths on the order of tens of centimetres to allow for complete mixing [70]. Therefore, microanalytical systems that have to find strategies for effective mixing, immobilization, separation, and transduction can take advantage of the apparent dual functionality of nanofibres and don’t have to use additional mixing structures when already using nanofibres as functional components in their systems. While expertise in electrospinning is required to spin and functionalize the fibres, a basic electrospinning system consists only of a syringe pump, a high voltage source, and a grounded collector plate and can be carried out in any lab setting. It is thus much simpler than most microfluidic fabrication methods. Additionally, fibres produced by electrospinning can be spun out of many different polymers and with many different functionalities [71,72,73]. Furthermore, the two-layer PVA micromixers described in this work produced an average outlet mixing index of between 0.29 and 0.36, corresponding to 71% and 64% fluid mixing, respectively. These mixing index values are comparable to or better than several previously reported passive micromixers, such as circular baffle, diamond obstacle, and split and recombine mixers, though it is less than the 90% mixing observed using zigzag mixing channels (Table 3). However, unlike these other micromixers, electrospun nanofibre mats can easily be further functionalized to allow for coupling of fluid mixing directly with analyte detection or sample preparation as demonstrated in our research group [21,23,24]. The increased surface area provided by the nanofibre mats would also increase the sensitivity of detection when compared to conventional, two-dimensional sensors due to an increase in binding sites within the device.

While electrospun fibre mats are a promising alternative to conventional micromixers, the electrospinning process depends on many different parameters (such as solution viscosity, ambient temperature and humidity, feed rate, applied voltage, etc.) which can make precise control of fibre morphology difficult. Further, because electrospinning typically uses a spinneret to produce ultrathin fibres, it can have a lower yield than would be ideal for some industrial applications [74]. Therefore, future work will seek to optimize the fibre formation process used to produce the fibre-based micromixers to allow for more precise control over fibre morphology and deposition. The use of temperature and humidity controlled spinning systems could be examined to reduce beading and better control solvent evaporation. Further, alternative collector plates could be used to allow for better fibre alignment. In particular, rotating drum [75], aligned electrode [76], and air-flow impedance [77] collector plates can be used to more precisely control fibre alignment and distribution. Finally, alternative fibre formation processes could be utilized to address the reproducibility and scalability of the electrospinning process including meltblowing, bi-component spinning, forcespinning and flashspinning [78]. Mahalingam et al. have developed a pressurised gyration fibre formation process that could be used for mass production of fibres such as those used in this work [79].

## 5. Conclusions

Multiple morphologies of electrospun poly(vinyl alcohol) and polystyrene fibres were incorporated into poly(methyl methacrylate) microchannels to study their ability to enhance fluid mixing. The most mixing was observed in the PVA nanofibre mats, which had a diameter of 450–550 nm. The PVA nanofibre mats had an inhomogeneous morphology, which is likely the cause of the increased fluid mixing when compared to the more homogeneous PS fibre mats. On the other hand, the PS fibres likely produce mixing due to their volumetric presence within the channel, acting much like traditional microstructure-based mixers. Further, the electrospun fibre mats can easily be functionalized to allow for sensitive analyte detection as well as fluid mixing. The reproducible mixing and relatively easy fabrication of electrospun fibre mats makes them a highly interesting functional component for microanalytical systems in which reaction, separation, immobilization, and transduction capabilities can be combined with their inherent passive mixing performance. Amazingly, they even outperform many passive mixers described in literature. As previously demonstrated in our lab, nanofibres can function as a concentration matrix [23] and as an immobilization matrix [42]—the added benefit of mixing produced by the nanofibres makes them thus an even more interesting new material to study for use in bioanalytical sensing systems.

## Figures and Tables

**Table 1:** Average mixing index in channels containing different PVA nanofibre mats. Each mixing index was calculated for channels with a flow rate of 1 µL/min. Values shown are calculated as the average mixing index of at least three channels.

Morphology	Mixing Index (inlet)	Standard Deviation	Mixing Index (outlet)	Standard Deviation	Difference from Control Outlet	Standard Deviation
**Control: No Fibres**	0.8	0.17	0.71	0.12	-	-
**3 mm 1 layer**	0.56	0.20	0.41	0.13	0.30	0.17
**3 mm 2 layer**	0.53	0.07	0.36	0.09	0.35	0.15
**5 mm 1 layer**	0.64	0.08	0.45	0.11	0.26	0.16
**5 mm 2 layer**	0.45	0.08	0.29	0.09	0.42	0.15
**10 mm 1 layer**	0.74	0.22	0.52	0.07	0.19	0.14
**10 mm 2 layer**	0.57	0.21	0.32	0.06	0.39	0.13

**Table 2:** Average mixing index in channels containing different PS fibre mats. Calculated for channels with a flow rate of 1 µL/min. Each mixing index is calculated as the average mixing index of at least three channels.

Morphology	Mixing Index (Inlet)	Standard Deviation	Mixing Index (Outlet)	Standard Deviation	Difference from Control Outlet Mixing Index	Standard Deviation
**Control: No Fibres**	0.80	0.17	0.71	0.12	-	-
**1 layer 12.5%**	0.84	0.12	0.64	0.17	0.07	0.21
**2 layer 12.5%**	0.77	0.05	0.56	0.09	0.15	0.15
**1 layer 15%**	0.87	0.02	0.78	0.05	−0.07	0.13
**2 layer 15%**	0.67	0.05	0.49	0.03	0.22	0.12
**1 layer 17.5%**	0.71	0.28	0.56	0.19	0.15	0.22
**2 layer 17.5%**	0.72	0.23	0.57	0.18	0.14	0.22

**Table 3:** Comparison of passive micromixers.

Mixer	Setting	Mixing Index	Reference
**Split and Recombine**	Re = 10	0.9	Ansari et al. [65]
	Re = 60	0.7	Ansari et al. [65]
**Diamond Obstacles**	Asymmetric Distribution	0.2	Bhagat et al. [57]
**Zigzag**		0.1	Jeon et al. [66]
**Circular Baffles**		0.3	Jeon et al. [66]
**PVA Nanofibres**	2 layer 5 mm	0.3	This work

## Supplementary Materials

The following are available online at http://www.mdpi.com/1424-8220/16/8/1238/s1: Figure S1: Schematic of a basic electrospinning apparatus; Figure S2: Schematic of hot embossing and bonding protocol; Figure S3: Confocal Z scan of a channel during mixing experiments; Figure S4: Thick nanofibre experiments; Figure S5: Outlet mixing index at different flow rates, Table S1: Multiple linear regression variables and their outcome (PVA fibres); Table S2: Multiple linear regression variables and their outcome for analyzing the effect of poly(vinyl alcohol) mat morphology; Figure S6: Flow profiles in channels containing PS fibre mats; Figure S7: Flow profiles of inlets of channels containing PS fibre mats; Figure S8: Flow profiles at outlets of channels containing PS fibre mats; Table S3: Multiple linear regression variables and their outcomes (PS fibres); Table S4: Multiple linear regression variables and their outcomes for analyzing the effects of polystyrene mat morphology on mixing index; Code S1: PVA analysis-effect of length and number of fibre mat layers; Code S2: PVA analysis-comparison of each PVA mat type; Code S3: PS Analysis-effect of polymer weight percent and number of layers; Code S4: PS analysis-comparison of each type of PS fibre mat.
