# Supplementary material for: Passive Mixing Capabilities of Micro- and Nanofibres When Used in Microfluidic Systems
Source: Sensors (Basel). 2016 Aug 5;16(8):1238. doi: 10.3390/s16081238 (PMC5017403; doi:10.3390/s16081238)
Supplement: Supplementary file 1 [file sensors-16-01238-s001.pdf]

Sensors **2016**, *16*, 1238; doi:10.3390/s16081238

Supplementary Materials: Passive Mixing Capabilities of Micro- and Nanofibres When Used in Microfluidic Systems

Lauren Matlock-Colangelo, Nicholas W. Colangelo, Christoph Fenzl, Margaret W. Frey and Antje J. Baeumner

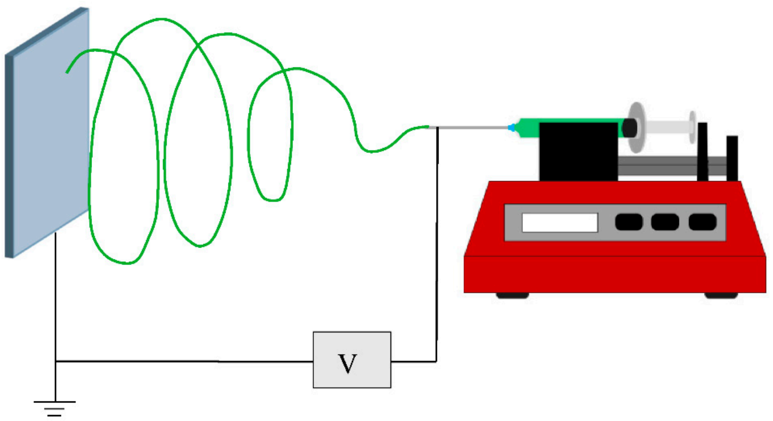

Figure S1. A typical electrospinning apparatus consisting of a syringe filled with a polymer spinning dope, a high voltage power source, a grounded collector plate, and a syringe pump.

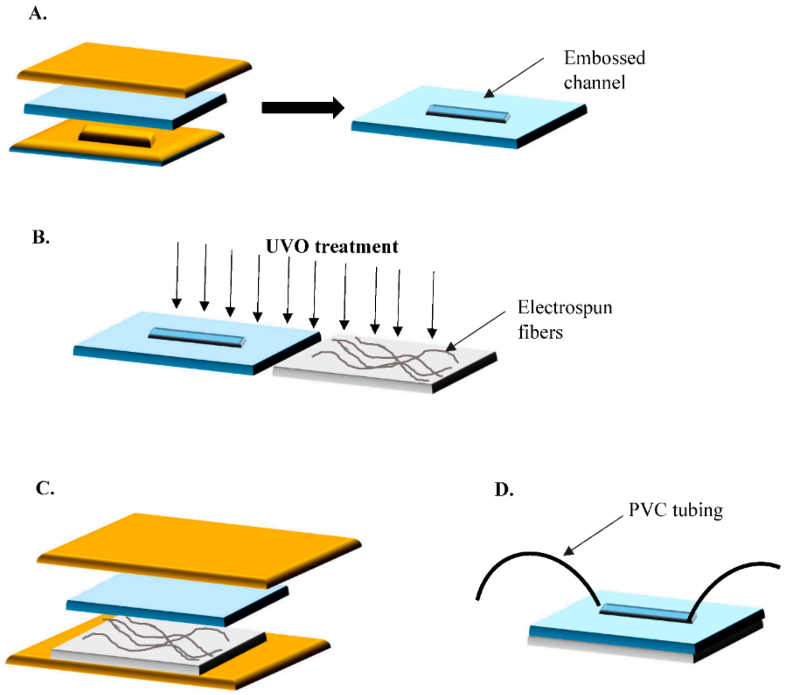

Figure S2. (**A**) Hot Embossing-A copper template with a raised channel structure is used to emboss the channel design in a piece of PMMA. The PMMA is sandwiched between the copper template and a blank piece of copper and placed on a Carver Laboratory Hot Press at 130 °C and 10,000 lbs (44,482 N) of force. Inlet at outlet holes are drilled at the ends of the channels; (**B**) UVO Treatment—The embossed PMMA and another piece of PMMA modified with electrospun fibers are both placed in a UV-ozone oven and treated to allow for UVO-assisted thermal bonding; (**C**) Bonding—The UVO-treated PMMA pieces are sandwiched together (with the fibers facing the embossed channels) and placed between two blank pieces of copper. The assembly is placed on the hot press at bonded together at a lower temperature and pressure than hot embossing; (**D**) Completed Device—Polyvinyl chloride tubing is glued into the inlet and outlet holes of the completed device.

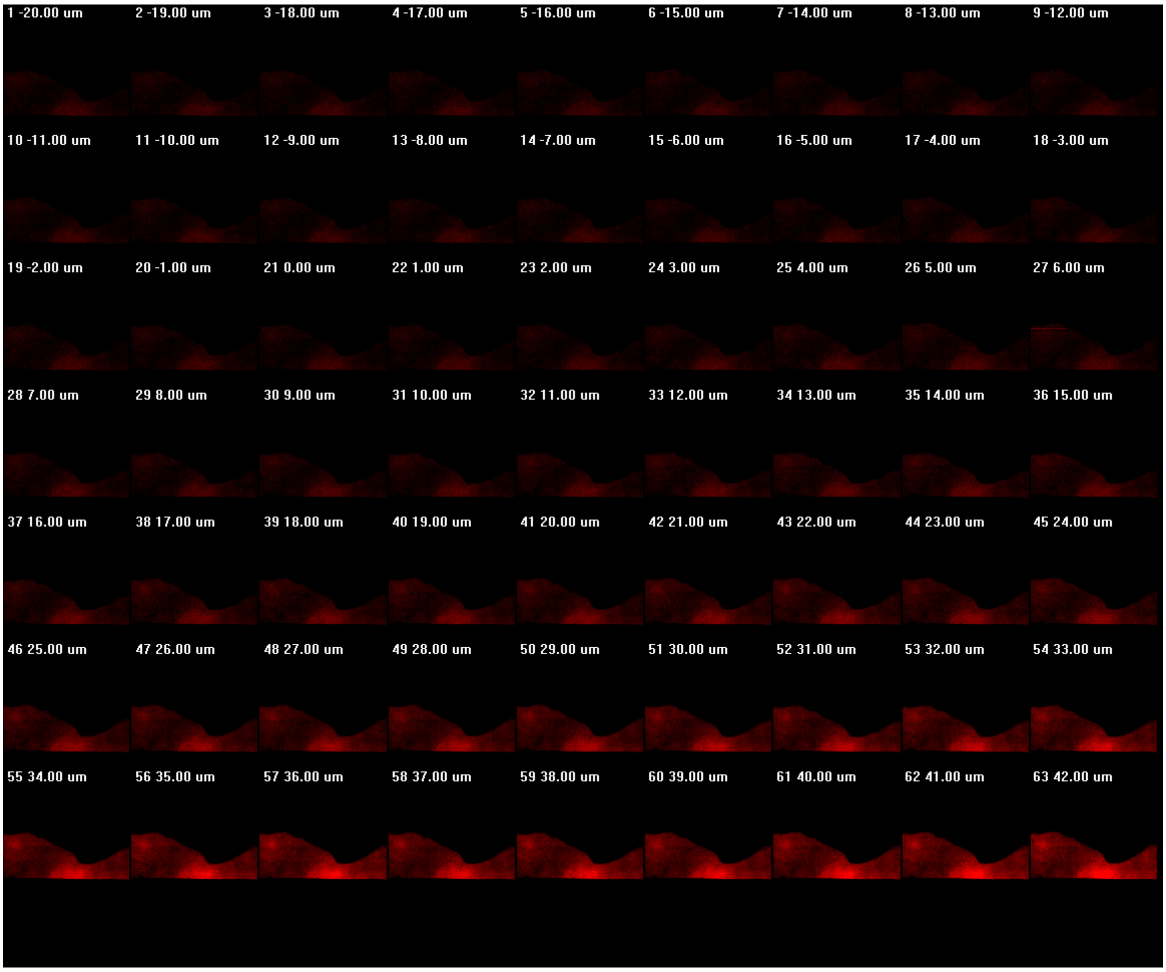

Figure S3. A confocal Z scan of a microfluidic channel during fluid mixing experiments. The flow profile does not change as the focal point moves in the z direction, indicating that it is consistent throughout the height of the microchannel. Channel images shown were taken at 1 µm intervals.

Data Analysis

We have added a step-by-step discussion of the calculation of the mixing index into the SI:

Data Analysis

The mixing index is calculated using the following formula:

$$mixing\ index = \sqrt{\frac{1}{N}\sum\left(\frac{I_K - I_O}{I_O}\right)^2}$$

I_K = intensity of an individual pixel in a column;

I_O = average intensity of all pixels in a column;

N = number of pixels in a column.

The mixing index for each channel is a value between 0 and 1, where a value of 0 indicates complete mixing within the channel. The mixing index was chosen to quantify the degree of mixing because it allowed us to compare our mixing results with the results from other labs.

When calculating the mixing index, a fluorescent microscopy image of the channel was taken during fluid flow. Images were taken either at the inlet (before the fiber mat) or at the outlet (after the fiber mat):

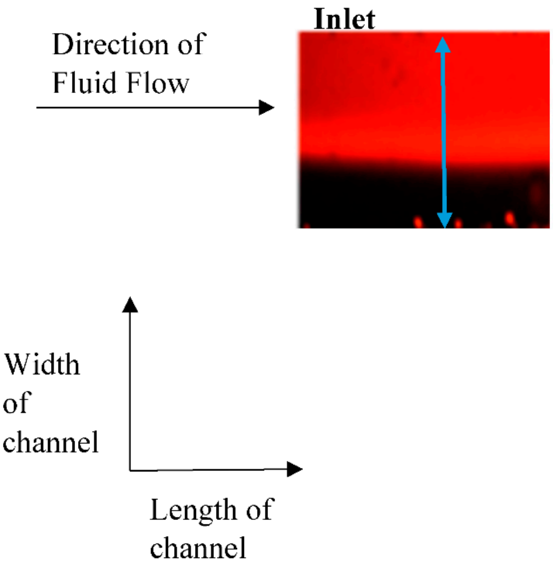

In order to calculate the mixing index, we first needed to get the intensity of each individual pixel in a column (blue arrow in above image) that extends along the width of the channel (these are our I_k values) as well as the average intensity of all the pixels in that column (this is our I_o value). The length of the column in pixels is our N value. This was done for 50 consecutive columns in each photo taken using the following ImageJ command:

```
run("ROI Manager...")
for (i=Xn; i<Xn+50;i++) {
makeLine (i,Y1,i,Y2);
roiManager("Add");
}
roiManager("Multi Plot");
```

where Xn is the x location of the first column being measured (where x is its location along the length of the channel) and Y1 and Y2 are the y coordinates of the top and bottom of the channel (along the width of the channel) that indicate where the column being measured:

Using this code, ImageJ produced a plot of the pixel intensity along each column as well as a list of the intensities for all the columns measured. An example of the pixel intensity plot for a single column is seen below:

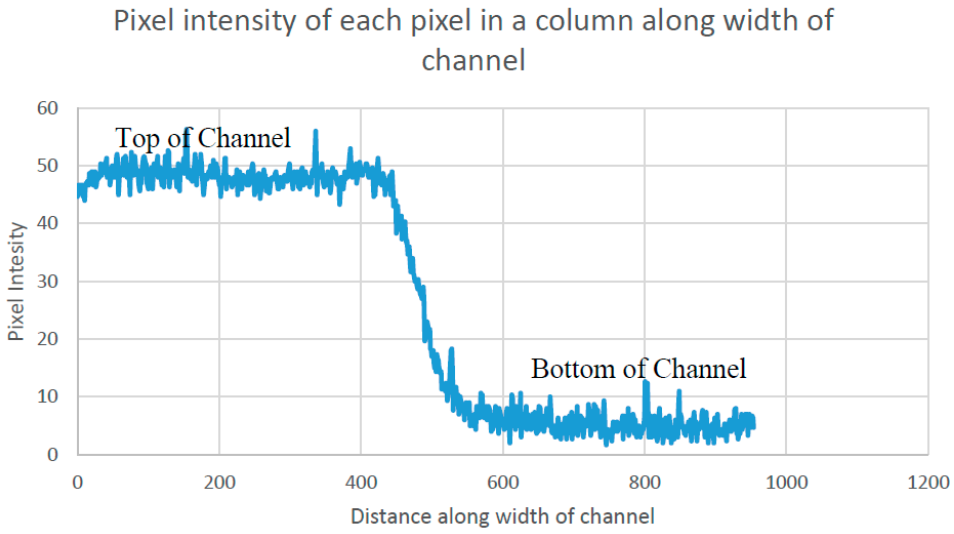

The average intensity (I_o) of the pixels in the column was calculated from the imageJ data and then the mixing index was calculated for each column. The mixing index values for each of the 50 columns were then averaged to get the average mixing index for that channel. Mixing indices are provided here with two decimal places to provide a more refined analyses of difference between the channels and mats analyzed.

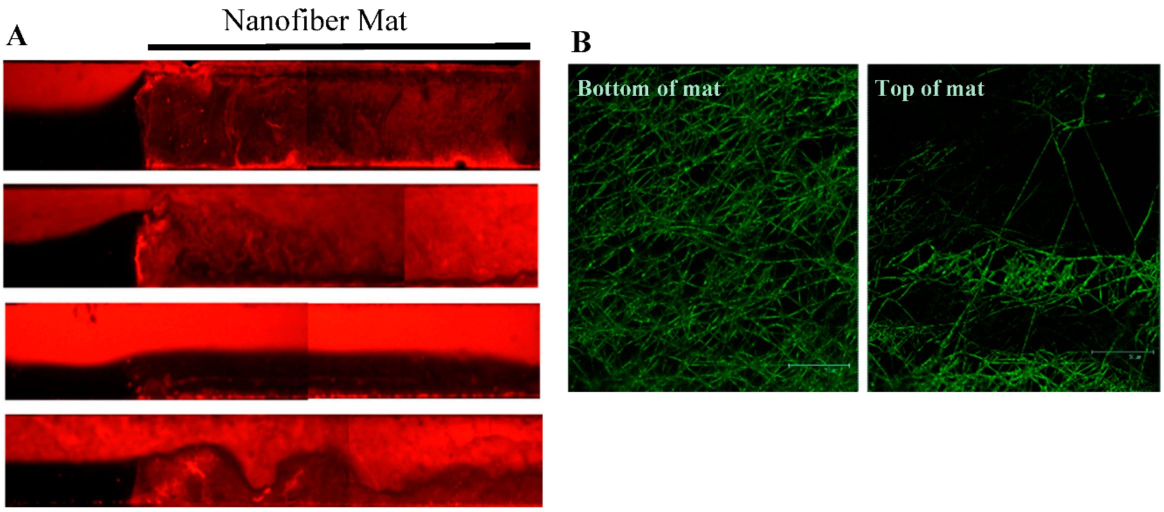

Figure S4. (**A**) Examples of flow profiles observed in thick nanofiber mat samples; (**B**) Variation in nanofiber porosity and distribution along thickness of nanofiber mats. Confocal microscopy.

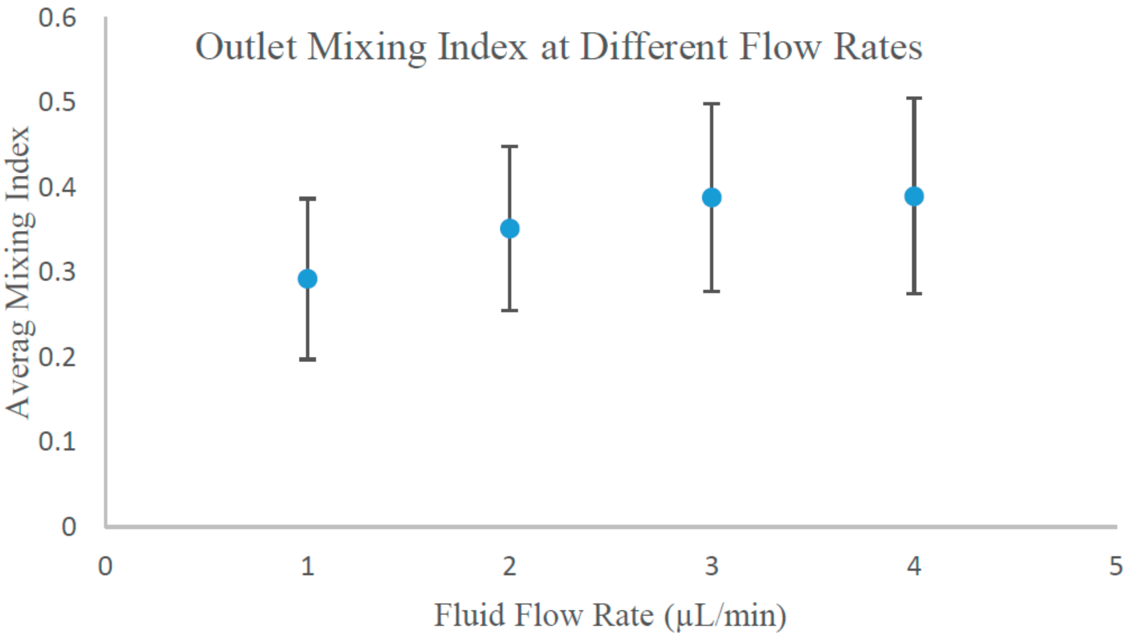

Figure S5. Outlet mixing index observed in 5 mm long two-layer mats at different flow rates. 3 channels were tested at each flow rate.

Table S1. Multiple linear regression variables and their outcome. The outlet mixing index values for each PVA mat morphology was compared to the outlet mixing index for empty control channels using linear regression. The linear regression controlled for the effect of flow rate on the mixing value. It was determined that mixing was significantly increased in all of the fiber mats, with the greatest mixing increase seen with the two-layer mats.

Variable	Significant within Family of Comparisons?	Average Change to Mixing Value	*p*-Value
3 mm, 1 Layer	Yes	−0.182	1.7234×10^{-5}
3 mm, 2 Layer	Yes	−0.285	2.3516×10^{-10}
5 mm, 1 Layer	Yes	−0.209	3.5847×10^{-7}
5 mm, 2 Layer	Yes	−0.344	4.4726×10^{-12}
10 mm, 1 Layer	Yes	−0.136	2.3049×10^{-3}
10 mm, 2 Layer	Yes	−0.340	6.6718×10^{-12}

Table S2. Multiple linear regression variables and their outcome for analyzing the effect of poly(vinyl alcohol) mat morphology. There were three families for comparison: (1) the different fiber mat lengths, (2) having two layers, and (3) the flow rate. Having two layers and flow rate were each considered to be significantly increased. However, the different fiber mat lengths did not appear to have an effect on mixing index.

Variable	Significant within Family of Comparisons?	Average Change to Mixing Index Value	p-Value
3 mm	No	-	
5 mm	No	-	Over all p = 0.24
10 mm	No	-	
2 Layers	Yes	−0.142	5.15×10^{-8}
Flow Rate	Yes	0.03 per (μL/s)	0.0045

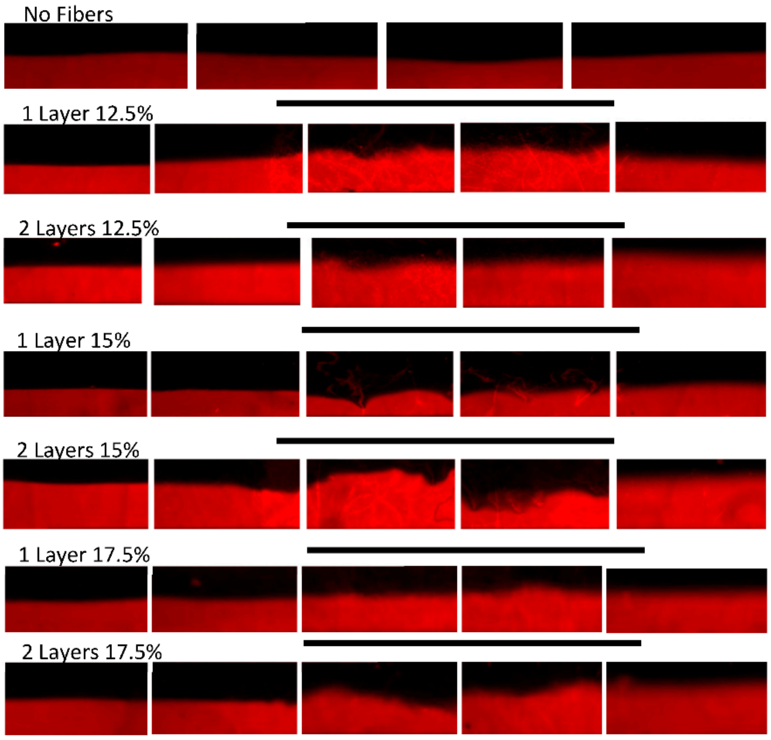

Figure S6. Flow profiles observed in channels containing PS fiber mats. Flow at 1 μL/min. Black lines indicate location of nanofiber mats within the channels. Leica Fluorescent Microscope. 5× objective.

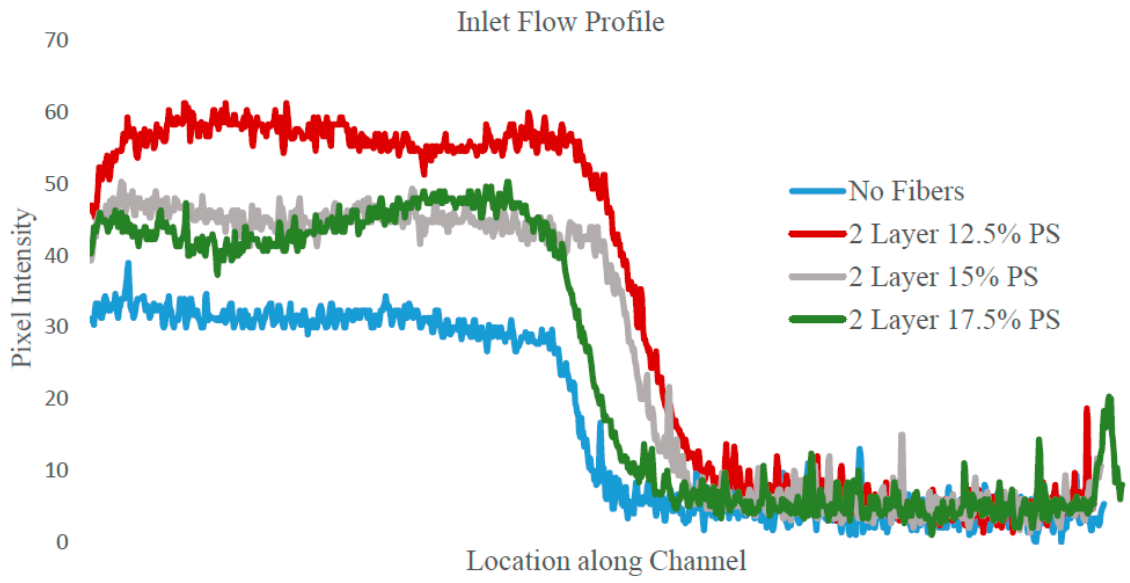

Figure S7. Flow profiles at inlets of channels containing different PS fiber morphologies. Flow rate was 1 μL/min.

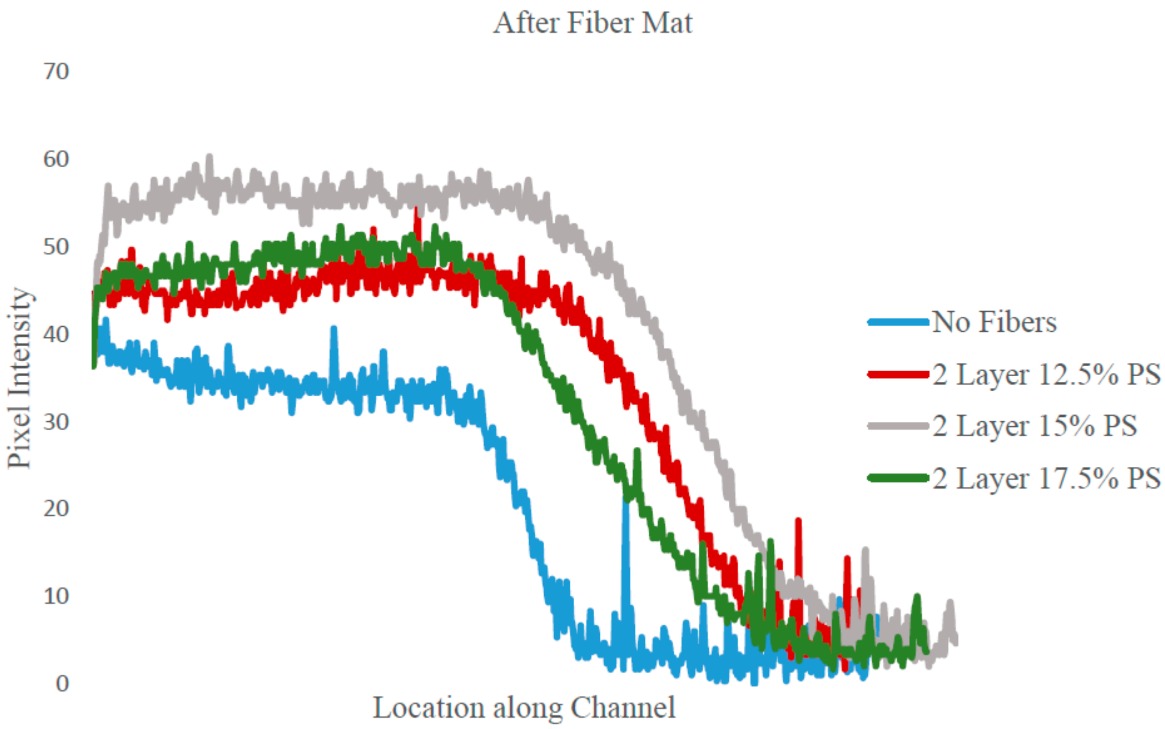

Figure S8. Flow profiles at outlets of channels containing different PS fiber morphologies. Flow rate was 1 μL/min.

Table S3. Multiple linear regression variables and their outcome. The different fiber mat types were considered as one family for comparison, and the flow rate another using linear regression. The linear regression controlled for the effect of flow rate on fluid mixing. 1 Layer 17.5%, 2 Layer 15%, and 2 Layer 17.5%, along with flow rate, were all considered to be significantly increased.

Variable	Significant within Family of Comparisons?	Average Change to Mixing Index Value	*p*-Value
1 Layer, 12.5%	No	-	-
1 Layer, 15%	No	-	-
1 Layer, 17.5%	Yes	−0.128	0.0018
2 Layer, 12.5%	No	-	-
2 Layer, 15%	Yes	−0.146	0.00046
2 Layer, 17.5%	Yes	−0.127	0.0021

Table S4. Multiple linear regression variables and their outcomes for analyzing effects of polystyrene mat morphology on mixing index. The effect of fiber diameter, fiber mat height, and flow rate on outlet mixing index was determined using multiple linear regression. The mixing increased with increasing fiber diameter, fiber mat height, and decreased with increasing flow rate.

Variable	Compared To	Significant within Family of Comparisons?	Average Change to Mixing Value	*p*-Value
15%	12.5%	No	-	0.956
17.5%	12.5%	Yes	−0.078	0.0077
17.5%	15%	Yes	−0.077	0.0090
2 Layers	1 Layer	Yes	−0.084	5.17×10^{-4}
Flow Rate	-	Yes	0.017 per (μL/min)	0.0481

MATLAB Code for Statistical Analysis of Fluid Mixing.

Code S1. PVA Analysis-Effect of Length and Number of Fiber Mat Layers

```
%Multiple Regression on PVA Data Separated by Length and Layer
    %Author: Lauren Colangelo and Nicholas Colangelo
    %Last Edit: 03-23-2015

    %As long as data input into the Table appropriately, everything should be taken care of between data sets except for the following,
    %which should be done for each new data set:
    %To do:
    %1) Make sure boxplots and other plots represent the data to be analyzed.
    %2) Make sure labels for plots are appropriate.
    %3) Make sure Holm Test is using the appropriate data from the SortData Table.
    %Clear memory of variables that may be used here:
      clear
    %Load the data:
      Data = readtable('C:\MatlabPVASep.xlsx');
      SortData = sortrows(Data,'Flow');
      SortData.Type = categorical(SortData.Type,'Ordinal',false);
      SortData.Length = categorical(SortData.Length,'Ordinal',false);
      SortData.Layer = categorical(SortData.Layer,'Ordinal',false);
      SortData.Channel = categorical(SortData.Channel,'Ordinal',false);

    %Box Plot:
      figure(1)
      subplot(2,2,1)
```

```
    boxplot(SortData.Mixing,SortData.Length)
    xh = xlabel('Length');
    yh = ylabel('Mixing Value');
    set([xh,yh],'fontweight','bold');
    set(gca,'XTick', 1:3)
    set(gca,'XTickLabel',{'3 mm', '5 mm', '10 mm'});

    subplot(2,2,2)
    boxplot(SortData.Mixing,SortData.Layer)
    xh = xlabel('Layer');
    yh = ylabel('Mixing Value');
    set([xh,yh],'fontweight','bold');

    subplot(2,2,3)
    boxplot(SortData.Mixing,SortData.Flow)
    xh = xlabel('Flow (\muL/sec)');
    yh = ylabel('Mixing Value');
    set([xh,yh],'fontweight','bold');
  %Regression:
    modelspec = 'Mixing ~ Length + Layer + Flow';
    lm = fitlm(table2dataset(SortData),modelspec);
    disp(lm);
    %Table of ANOVA Results:
    disp(anova(lm));
    %Show Number of Outliers as Determined by Cook's Distance >= 3*MeanCooksDistance:
    Cook = sum(lm.Diagnostics.CooksDistance./mean(lm.Diagnostics.CooksDistance) >= 3);
    fprintf('Number of Values above Cook''s Distance %f', Cook)
  %Obtain Parameters for Holm Test from the Regression
    %MSres
      MSres = double(lm.MSE);
    %Degrees of Freedom
      DF = double(lm.DFE);
  %Length
    %Number of each length:
    for i = 1:max(double(SortData.Length)) %different types
      n(i) = sum(double(SortData.Length) == i);
    end %i
    %Input the number of types, less one (So it doesn't compare values against themselves)
    for i = 1:(max(double(SortData.Length)-1))
      %Input the total number of types, so it can make all the comparisons
      for j = (i+1):max(double(SortData.Length))
        %Comparison to Control
        if i  == 1
          t(i,j) = (lm.Coefficients.Estimate(j) - 0)/ sqrt(MSres*(1/n(j)+1/n(i)));
        %Between types comparison
        else
          t(i,j)       =       (lm.Coefficients.Estimate(j)       -       lm.Coefficients.Estimate(i))/
sqrt(MSres*(1/n(j)+1/n(i)));
        end %if i
      end %j
    end %i
      %Now we take the t values to p values
      p1 = 2*(1-tcdf(abs(t),DF));
```

```
        %Organize p-values
        Sorted1 = sort(reshape(p1,1,size(t,1)*size(t,2)));
        %Holm test p-value for comparison with uncorrected p-value of p = 0.05:
          %Calculate number of comparisons:
          Comparisons = 0;
          i = 0;
          while i < max(double(SortData.Length))
            for i = 1:max(double(SortData.Length))
              Comparisons = Comparisons + (i-1);
            end %for i
          end %while i
          %Determine Significant Terms
          detect1 = 0;
          for i = 1:Comparisons
            pcomp = 0.05/(Comparisons-i+1);
            if Sorted1(i) <= pcomp
              Sig1(i) = 1;
              detect1 = 1;
            else
                break
            end
        end %i
        %Display p values in p matrix that are significant
        if detect1 == 1
          fprintf('\np1 matrix is:\n')
          format short e
          disp(p1.*(p1<=Sorted1(length(Sig1))))
        else
          fprintf('\np1 matrix has no significant values.\n')
        end %if
    %Layer
      %A lot of this code is unnecessary as there is only two layers to
      %compare.
      %Number of each Layer:
      for i = 1:max(double(SortData.Layer)) %different types
        n2(i) = sum(double(SortData.Layer) == i);
      end %i
        %Input the number of types, less one (So it doesn't compare values against themselves)
      for i = 1:(max(double(SortData.Layer)-1))
        %Input the total number of types, so it can make all the comparisons
        for j = (i+1):max(double(SortData.Layer))
          %Comparison to Control
          %Note: Accessing the coefficient array must account for Length variables
          if i  == 1
            t2(i,j)   =   (lm.Coefficients.Estimate(max(double(SortData.Length))-1+j)   -   0)/
sqrt(MSres*(1/n2(j)+1/n2(i)));
          %Between types comparison
          else
            t2(i,j)   =   (lm.Coefficients.Estimate(max(double(SortData.Length))-1+j)   -
lm.Coefficients.Estimate(i))/ sqrt(MSres*(1/n2(j)+1/n2(i)));
          end %if i
        end %j
```

```
    end %i
      %Now we take the t values to p values
      p2 = 2*(1-tcdf(abs(t2),DF));
      %Organize p-values
      Sorted2 = sort(reshape(p2,1,size(t2,1)*size(t2,2)));
      %Holm test p-value for comparison with uncorrected p-value of p=0.05:
        %Calculate number of comparisons:
        Comparisons2 = 0;
        i = 0;
        while i < max(double(SortData.Layer))
          for i = 1:max(double(SortData.Layer))
            Comparisons2 = Comparisons2 + (i-1);
          end %for i
        end %while i
        %Determine Significant Terms
        detect2 = 0;
        for i = 1:Comparisons2
          pcomp = 0.05/(Comparisons2-i+1);
          if Sorted2(i) <= pcomp
            Sig2(i) = 1;
            detect2 = 1;
          else
              break
          end %p(i)
        end %i
      %Display p values in p matrix that are significant
      if detect2 == 1
        fprintf('\np2 matrix is:\n')
        format short e
        disp(p2.*(p2<=Sorted2(length(Sig2))))
      else
        fprintf('\np2 matrix has no significant values.\n')
      end %if

  %Residuals
  figure(2)
    gscatter(double(SortData.Type),lm.Residuals.Raw,SortData.Flow)
    yh = ylabel('Residuals');
    xh = xlabel('Types');
    legh = legend;
    htitle = get(legh,'Title');
    set(htitle,'String','Flow (\muL/sec)')
    set([xh,yh],'fontweight','bold');
    set(gca,'XTick', 1:6)
    set(gca,'XTickLabel',{'3 mm 1 Layer', '3 mm 2 Layer', '5 mm 1 Layer', '5 mm 2 Layer', '10 mm
1 Layer', '10 mm 2 Layer'})

  %Test Normality
  figure(3)
  normplot(lm.Residuals.Standardized)
  xh = xlabel('Standardized Residuals');
  yh = ylabel('Normal Probability Scale');
  set([xh,yh],'fontweight','bold');
```

Code S2. PVA Analysis-Comparison of each PVA mat type

```
%Multiple Regression on PVA by Type
%Author: Lauren Colangelo and Nicholas Colangelo
%Last Edit: 03-23-2015
%As long as data input into the Table appropriately, everything should be taken care of between data sets except for the following,
%which should be done for each new data set:
%To do:
%1) Make sure boxplots and other plots represent the data to be analyzed.
%2) Make sure labels for plots are appropriate.
%3) Make sure Holm Test is using the appropriate data from the SortData Table.

%Clear memory of variables that may be used here:
  clear
%Load the data:
  Data = readtable('C:\MatlabPVAType.xlsx');
  SortData = sortrows(Data,'Flow');
  SortData.Type = categorical(SortData.Type,'Ordinal',false);
  SortData.Channel = categorical(SortData.Channel,'Ordinal',false);
%Box Plot:
  figure(1)
  subplot(2,1,1)
  boxplot(SortData.Mixing,SortData.Type)
  xh = xlabel('Type');
  yh = ylabel('Mixing Value');
   set([xh,yh],'fontweight','bold');
  set(gca,'XTick', 1:7)
  set(gca,'XTickLabel',{'Control', '3 mm 1 Layer', '3 mm 2 Layer', '5 mm 1 Layer', '5 mm 2 Layer', '10 mm 1 Layer', '10 mm 2 Layer'});
  subplot(2,1,2)
  boxplot(SortData.Mixing,SortData.Flow)
  xh = xlabel('Flow (\muL/min)');
  yh = ylabel('Mixing Value');
  set([xh,yh],'fontweight','bold');
%Regression:
  modelspec = 'Mixing ~ Type + Flow';
  lm = fitlm(table2dataset(SortData),modelspec);
  disp(lm);
  %Table of ANOVA Results:
  disp(anova(l m));
  %Show Number of Outliers as Determined by Cook's Distance >= 3*MeanCooksDistance:
  Cook = sum(lm.Diagnostics.CooksDistance./mean(lm.Diagnostics.CooksDistance) >= 3);
  fprintf('Number of Values above Cook''s Distance %f', Cook)
%Obtain Parameters for Holm Test from the Regression
  %TBL(3,3) = MSres
    MSres = double(lm.MSE);
  %TBL(3,2) = Degrees of Freedom
    DF = double(lm.DFE);
  %Number of each type:
  for i = 1:max(double(SortData.Type)) %different types
    n(i) = sum(double(SortData.Type) == i);
  end %i
```

```
%Holm Test
    %Input the number of types, less one (So it doesn't compare values against themselves)
    for i = 1:(max(double(SortData.Type)-1))
        %Input the total number of types, so it can make all the comparisons
        for j = (i+1):max(double(SortData.Type))
            %Comparison to Control
            if i  == 1
                t(i,j) = (lm.Coefficients.Estimate(j) - 0)/ sqrt(MSres*(1/n(j)+1/n(i)));
            %Between types comparison
            else
                t(i,j) = (lm.Coefficients.Estimate(j) - lm.Coefficients.Estimate(i))/ sqrt(MSres*(1/n(j)+1/n(i)));
            end %if i
        end %j
    end %i
    %Now we take the t values to p values
    p = 2*(1-tcdf(abs(t),DF));
    %Organize p-values
    Sorted = sort(reshape(p,1,size(t,1)*size(t,2)));
    %Holm test p-value for comparison with uncorrected p-value of p=0.05:
        %Calculate number of comparisons:
        Comparisons = 0;
        i = 0;
        while i < max(double(SortData.Type))
            for i = 1:max(double(SortData.Type))
                Comparisons = Comparisons + (i-1);
            end %for i
        end %while i
        %Determine Significant Terms
        detect = 0;
        for i = 1:Comparisons
            pcomp = 0.05/(Comparisons-i+1);
            if Sorted(i) <= pcomp
                Sig(i) = 1;
                detect = 1;
            else
                    break
            end %if Sorted
        end %if i
        %Display p values in p matrix that are significant
        if detect == 1
            fprintf('\np matrix is:\n')
            format short e
            disp(p.*(p<=Sorted(length(Sig))))
        else
            fprintf('\np1 matrix has no significant values.\n')
        end %if
%Residuals

figure(2)
    gscatter(double(SortData.Type),lm.Residuals.Raw,SortData.Flow)
    yh = ylabel('Residuals');
    xh = xlabel('Types');
```

```
legh = legend;
htitle = get(legh,'Title');
set(htitle,'String','Flow (\muL/min)')
set([xh,yh],'fontweight','bold');
set(gca,'XTick', 1:7)
set(gca,'XTickLabel',{'Control', '3 mm 1 Layer', '3 mm 2 Layer', '5 mm 1 Layer', '5 mm 2 Layer',
'10 mm 1 Layer', '10 mm 2 Layer'})

%Test Normality with a Normal Probability Plot
figure(3)
normplot(lm.Residuals.Standardized)
xh = xlabel('Standardized Residuals');
yh = ylabel('Normal Probability Scale');
set([xh,yh],'fontweight','bold');
```

Code S3. PS Analysis- Effect of polymer weight percent and number of layers

```
%Multiple Regression on Polystyrene Data Separated by Weight and Layer
%Author: Lauren Colangelo and Nicholas Colangelo
%Last Edit: 03-23-2015
%As long as data input into the Table appropriately, everything should be taken care of between
data sets except for the following,
%which should be done for each new data set:
%To do:
%1) Make sure boxplots and other plots represent the data to be analyzed.
%2) Make sure labels for plots are appropriate.
%3) Make sure Holm Test is using the appropriate data from the SortData Table.

%Clear memory of variables that may be used here:
clear
%Load the data:
Data = readtable('C:\MatlabPolyStySep.xlsx');
SortData = sortrows(Data,'Flow');
SortData.Type = categorical(SortData.Type,'Ordinal',false);
SortData.Weight = categorical(SortData.Weight,'Ordinal',false);
SortData.Layer = categorical(SortData.Layer,'Ordinal',false);
SortData.Channel = categorical(SortData.Channel,'Ordinal',false);

%Box Plot the data:
figure(1)

subplot(2,2,1)
boxplot(SortData.Mixing,SortData.Weight)
xh = xlabel('Weight');
yh = ylabel('Mixing Value');
set([xh,yh],'fontweight','bold');
set(gca,'XTick', 1:3)
set(gca,'XTickLabel',{'12.5%', '15%', '17.5%'});
subplot(2,2,2)
boxplot(SortData.Mixing,SortData.Layer)
xh = xlabel('Layer');
yh = ylabel('Mixing Value');
set([xh,yh],'fontweight','bold');
subplot(2,2,3)
boxplot(SortData.Mixing,SortData.Flow)
```

```
        xh = xlabel('Flow (\muL/sec)');
        yh = ylabel('Mixing Value');
        set([xh,yh],'fontweight','bold');

    %Regression:
      modelspec = 'Mixing ~ Weight + Layer + Flow';
      lm = fitlm(table2dataset(SortData),modelspec);
      disp(lm);
      %Table of ANOVA Results:
      disp(anova(lm));
      %Show Number of Outliers as Determined by Cook's Distance >= 3*MeanCooksDistance:
      Cook = sum(lm.Diagnostics.CooksDistance./mean(lm.Diagnostics.CooksDistance) >= 3);
      fprintf('Number of Values above Cook''s Distance %f', Cook)
    %Obtain Parameters for Holm Test from the Regression
      %MSres
        MSres = double(lm.MSE);
      %Degrees of Freedom
        DF = double(lm.DFE);

    %Weight Percentage
      %Number of each weight percentage:
      for i = 1:max(double(SortData.Weight)) %different types
        n(i) = sum(double(SortData.Weight) == i);
      end %i
      %Input the number of types, less one (So it doesn't compare values against themselves)
      for i = 1:(max(double(SortData.Weight)-1))
        %Input the total number of types, so it can make all the comparisons
        for j = (i+1):max(double(SortData.Weight))
          %Comparison to Control
          if i  == 1
            t(i,j) = (lm.Coefficients.Estimate(j) - 0)/ sqrt(MSres*(1/n(j)+1/n(i)));
          %Between types comparison
          else
            t(i,j)     =     (lm.Coefficients.Estimate(j)     -     lm.Coefficients.Estimate(i))/
sqrt(MSres*(1/n(j)+1/n(i)));
          end %if i
        end %j
      end %i
        %Now we take the t values to p values
        p1 = 2*(1-tcdf(abs(t),DF));
        %Organize p-values
        Sorted1 = sort(reshape(p1,1,size(t,1)*size(t,2)));
        %Holm test p-value for comparison with uncorrected p-value of p=0.05:
          %Calculate number of comparisons:
          Comparisons = 0;
          i = 0;
          while i < max(double(SortData.Weight))
            for i = 1:max(double(SortData.Weight))
              Comparisons = Comparisons + (i-1);
            end %for i
          end %while i
          %Determine Significant Terms
          detect1 = 0;
```

```
        for i = 1:Comparisons
            pcomp = 0.05/(Comparisons-i+1);
            if Sorted1(i) <= pcomp
                Sig1(i) = 1;
                detect1 = 1;
            else
                    break
            end
        end %i
        %Display p values in p matrix that are significant
        if detect1 == 1
            fprintf('\np1 matrix is:\n')
            disp(p1.*(p1<=Sorted1(length(Sig1))))
            format short e
        else
            fprintf('\np1 matrix has no significant values.\n')
        end %if

%Layer
    %A lot of this code is unnecessary as there is only two layers to
    %compare.
    %Number of each Layer:
    for i = 1:max(double(SortData.Layer)) %different types
        n2(i) = sum(double(SortData.Layer) == i);
    end %i
        %Input the number of types, less one (So it doesn't compare values against themselves)
    for i = 1:(max(double(SortData.Layer)-1))
        %Input the total number of types, so it can make all the comparisons
        for j = (i+1):max(double(SortData.Layer))
            %Comparison to Control
            %Note: Accessing the coefficient array must account for weight variables
            if i  == 1
                t2(i,j)    =    (lm.Coefficients.Estimate(max(double(SortData.Weight))-1+j)    -    0)/
sqrt(MSres*(1/n2(j)+1/n2(i)));
            %Between types comparison
            else
                t2(i,j)    =    (lm.Coefficients.Estimate(max(double(SortData.Weight))-1+j)    -
lm.Coefficients.Estimate(i))/ sqrt(MSres*(1/n2(j)+1/n2(i)));
            end %if i
        end %j
    end %i
        %Now we take the t values to p values
        p2 = 2*(1-tcdf(abs(t2),DF));
        %Organize p-values
        Sorted2 = sort(reshape(p2,1,size(t2,1)*size(t2,2)));
        %Holm test p-value for comparison with uncorrected p-value of p=0.05:
            %Calculate number of comparisons:
            Comparisons2 = 0;
            i = 0;
            while i < max(double(SortData.Layer))
                for i = 1:max(double(SortData.Layer))
                    Comparisons2 = Comparisons2 + (i-1);
                end %for i
```

```
        end %while i
        %Determine Significant Terms
        detect2 = 0;
        for i = 1:Comparisons2
          pcomp = 0.05/(Comparisons2-i+1);
          if Sorted2(i) <= pcomp
            Sig2(i) = 1;
            detect2 = 1;
          else
              break
          end %p(i)
        end %i
      %Display p values in p matrix that are significant
      if detect2 == 1
        fprintf('\np2 matrix is:\n')
        format short e
        disp(p2.*(p2<=Sorted2(length(Sig2))))
      else
        fprintf('\np2 matrix has no significant values.\n')
      end %if
  %Residuals
  figure(2)
    gscatter(double(SortData.Type),lm.Residuals.Raw,SortData.Flow)
    yh = ylabel('Residuals');
    xh = xlabel('Types');
    legh = legend;
    htitle = get(legh,'Title');
    set(htitle,'String','Flow (\muL/sec)')
    set([xh,yh],'fontweight','bold');
    set(gca,'XTick', 1:6)
    set(gca,'XTickLabel',{'3 mm 1 Layer', '3 mm 2 Layer', '5 mm 1 Layer', '5 mm 2 Layer', '10 mm 1 Layer', '10 mm 2 Layer'})

  %Test Normality
  figure(3)
    normplot(lm.Residuals.Standardized)
    xh = xlabel('Standardized Residuals');
    yh = ylabel('Normal Probability Scale');
    set([xh,yh],'fontweight','bold');
```

Code S4. PS Analysis- Comparison of each type of PS fiber mat

```
  %Multiple Regression on Polystyrene Data by Type
  %Author: Lauren Colangelo and Nicholas Colangelo
  %Last Edit: 03-23-2015

  %As long as data input into the Table appropriately, everything should be taken care of between data sets except for the following,

  %which should be done for each new data set:
  %To do:
  %1) Make sure boxplots and other plots represent the data to be analyzed.
  %2) Make sure labels for plots are appropriate.
  %3) Make sure Holm Test is using the appropriate data from the SortData Table.
```

```
%Clear memory of variables that may be used here:
   clear
%Load the data:
   Data = readtable('C:\MatlabPolyStyType.xlsx');
   SortData = sortrows(Data,'Flow');
   SortData.Type = categorical(SortData.Type,'Ordinal',false);
   SortData.Channel = categorical(SortData.Channel,'Ordinal',false);

%Box Plot:
   figure(1)
   subplot(2,1,1)
   boxplot(SortData.Mixing,SortData.Type)
   xh = xlabel('Type');
   yh = ylabel('Mixing Value');
    set([xh,yh],'fontweight','bold');
   set(gca,'XTick', 1:7)
   set(gca,'XTickLabel',{'Control', '1 Layer 12.5%', '1 Layer 15%', '1 Layer 17.5%', '2 Layer 12.5%',
'2 Layer 15%', '2 Layer 17.5%'});

   subplot(2,1,2)
   boxplot(SortData.Mixing,SortData.Flow)
   xh = xlabel('Flow (\muL/min)');
   yh = ylabel('Mixing Value');
   set([xh,yh],'fontweight','bold');
%Regression:
   modelspec = 'Mixing ~ Type + Flow';
   lm = fitlm(table2dataset(SortData),modelspec);
   disp(lm);
   %Table of ANOVA Results:
   disp(anova(lm));
   %Show Number of Outliers as Determined by Cook's Distance >= 3*MeanCooksDistance:
   Cook = sum(lm.Diagnostics.CooksDistance./mean(lm.Diagnostics.CooksDistance) >= 3);
   fprintf('Number of Values above Cook''s Distance %f', Cook)
%Obtain Parameters for Holm Test from the Regression
   %TBL(3,3) = MSres
      MSres = double(lm.MSE);
   %TBL(3,2) = Degrees of Freedom
      DF = double(lm.DFE);
   %Number of each type:
   for i = 1:max(double(SortData.Type)) %different types
      n(i) = sum(double(SortData.Type) == i);
   end %i

%Holm Test
   %Input the number of types, less one (So it doesn't compare values against themselves)
   for i = 1:(max(double(SortData.Type)-1))
      %Input the total number of types, so it can make all the comparisons
      for j = (i+1):max(double(SortData.Type))
         %Comparison to Control
         if i  == 1
            t(i,j) = (lm.Coefficients.Estimate(j) - 0)/ sqrt(MSres*(1/n(j)+1/n(i)));
         %Between types comparison
         else
```

```
                t(i,j)    =    (lm.Coefficients.Estimate(j)    -    lm.Coefficients.Estimate(i))/
sqrt(MSres*(1/n(j)+1/n(i)));
            end %if i
        end %j
    end %i
    %Now we take the t values to p values
    p = 2*(1-tcdf(abs(t),DF));
    %Organize p-values
    Sorted = sort(reshape(p,1,size(t,1)*size(t,2)));
    %Holm test p-value for comparison with uncorrected p-value of p=0.05:
        %Calculate number of comparisons:
        Comparisons = 0;
        i = 0;
        while i < max(double(SortData.Type))
            for i = 1:max(double(SortData.Type))
                Comparisons = Comparisons + (i-1);
            end %for i
        end %while i
        %Determine Significant Terms
        detect = 0;
        for i = 1:Comparisons
            pcomp = 0.05/(Comparisons-i+1);
            if Sorted(i) <= pcomp
                Sig(i) = 1;
                detect = 1;
            else
                    break
            end %if Sorted
        end %if i
        %Display p values in p matrix that are significant
        if detect == 1
            fprintf('\np matrix is:\n')
            format short e
            disp(p.*(p<=Sorted(length(Sig))))
        else
            fprintf('\np1 matrix has no significant values.\n')
        end %if

    %Residuals
    figure(2)
        gscatter(double(SortData.Type),lm.Residuals.Raw,SortData.Flow)
        yh = ylabel('Residuals');
        xh = xlabel('Types');
        legh = legend;
        htitle = get(legh,'Title');
        set(htitle,'String','Flow (\muL/min)')
        set([xh,yh],'fontweight','bold');
        set(gca,'XTick', 1:7)
        set(gca,'XTickLabel',{'Control', '1 Layer 12.5%', '1 Layer 15%', '1 Layer 17.5%', '2 Layer 12.5%',
'2 Layer 15%', '2 Layer 17.5%'})

    %Test Normality with a Normal Probability Plot
    figure(3)
```

```
normplot(lm.Residuals.Standardized)
xh = xlabel('Standardized Residuals');
yh = ylabel('Normal Probability Scale');
set([xh,yh],'fontweight','bold');
```
